# Integrated Metabolomics and Targeted Gene Transcription Analysis Reveal Global Bacterial Antimonite Resistance Mechanisms

**DOI:** 10.3389/fmicb.2021.617050

**Published:** 2021-01-28

**Authors:** Jingxin Li, Yuxiao Zhang, Xing Wang, Seth T. Walk, Gejiao Wang

**Affiliations:** ^1^State Key Laboratory of Agricultural Microbiology, College of Life Science and Technology, Huazhong Agricultural University, Wuhan, China; ^2^Department of Microbiology and Immunology, Montana State University, Bozeman, MT, United States

**Keywords:** *Agrobacterium tumefaciens*, Sb(III) resistance, global response, metabolomics, proteomics

## Abstract

Antimony (Sb)-resistant bacteria have potential applications in the remediation of Sb-contaminated sites. However, the effect of Sb(III) exposure on whole-cell metabolic change has not been studied. Herein, we combined untargeted metabolomics with a previous proteomics dataset and confirmatory gene transcription analysis to identify metabolic responses to Sb(III) exposure in *Agrobacterium tumefaciens* GW4. Dynamic changes in metabolism between control and Sb(III)-exposed groups were clearly shown. KEGG pathway analysis suggested that with Sb(III) exposure: (1) the branching pathway of gluconeogenesis is down-regulated, resulting in the up-regulation of pentose phosphate pathway to provide precursors of anabolism and NADPH; (2) glycerophospholipid and arachidonic acid metabolisms are down-regulated, resulting in more acetyl-CoA entry into the TCA cycle and increased capacity to produce energy and macromolecular synthesis; (3) nucleotide and fatty acid synthesis pathways are all increased perhaps to protect cells from DNA and lipid peroxidation; (4) nicotinate metabolism increases which likely leads to increased production of co-enzymes (e.g., NAD^+^ and NADP^+^) for the maintenance of cellular redox and Sb(III) oxidation. Expectedly, the total NADP^+^/NADPH content, total glutathione, and reduced glutathione contents were all increased after Sb(III) exposure in strain GW4, which contribute to maintaining the reduced state of the cytoplasm. Our results provide novel information regarding global bacterial responses to Sb(III) exposure from a single gene level to the entire metabolome and provide specific hypotheses regarding the metabolic change to be addressed in future research.

## Introduction

Antimony (Sb) is both a widely used metalloid and a public health threat (Wilson et al., 2010; Herath et al., 2017). The abundance of Sb is extremely low in the earth’s crust, but levels dramatically accumulate (up to 5000 mg/kg) in certain contaminated sites due to industrial emissions, mining, and smelting (He, 2007; Guo et al., 2009; He et al., 2012). The safety standard set by the World Health Organization (WHO, 2003) for Sb in drinking water is 5 μg/L (parts-per-billion, ppb), and many people in countries around the world drink water above this standard. Toxic Sb-containing compounds can enter human circulation at multiple points, including the airway (respiratory tract), digestive tract (ingestion), and/or contact with skin (Li et al., 2018). Chronic exposures are associated with a variety of pathologies, including increased risk of cardiovascular disease and cancer, and high-level, acute exposures can result in death.

Antimony can also be toxic to microorganisms, which have evolved a variety of strategies to resist its effects (Kulp et al., 2014; Terry et al., 2015; Li et al., 2016). For example, bacterial oxidation transforms a more toxic form, antimonite Sb(III), to a less toxic form, antimonate Sb(V), making this a detoxification process with respect to both bacterial cells and the environment (Filella et al., 2007). Given the ability of microbes to control important Sb detoxifying chemistries, a better understanding of microbe-Sb interactions (oxidation and other resistance mechanisms) could lead to more effective bioremediation efforts.

Studies on Sb(III) oxidation and resistance, including our own (Li et al., 2015; Li et al., 2016), have identified some of the key metabolic pathways involved. For example, the *ars* operon, which is responsible for arsenite As(III) resistance (Carlin et al., 1995), was shown to be involved in Sb(III) resistance. A product of this operon, the As(III)/Sb(III) carrier protein, ArsB, was also shown to catalyze Sb(III) efflux coupled with the hydrolysis of ATP in the presence of the ATPase, ArsA (Meng et al., 2004). Likewise, there is evidence that the transcriptional repressor, ArsR, and arsenate As(V) reductase, ArsC, are associated with Sb(III) resistance (Rosen, 1995), although their exact role(s) remain to be determined. In contrast to As(III) oxidation, which mechanism has been well studied in different genera of As(III)-oxidizing bacteria and archaea (Liu et al., 2012; Wang et al., 2015a; Shi et al., 2020), mechanism of Sb(III) oxidation has only recently been clarified in Sb(III)-oxidizing bacterium *Agrobacterium tumefaciens* (Li et al., 2015; Li et al., 2017a). We showed that Sb(III) oxidation is not a single enzymatic reaction in *Agrobacterium tumefaciens*, and that both the As(III) oxidase, AioAB, and Sb(III) oxidase, AnoA, participate in this pathway (Li et al., 2015; Wang et al., 2015b). In addition, we found that Sb(III)-driven oxidative stress, primarily via Sb(III)-induced upregulation of superoxide dismutase, *sod*, and catalase, *katA*, leads to non-enzymatic oxidation of Sb(III) (Li et al., 2017a). Clearly, broad microbial cellular responses to Sb are important to consider, but these aspects have not been adequately addressed so far.

Omics approaches have been applied to Sb(III)-exposed microbes, but have primarily focused on pathogenic *Leishmania spp.* because of their high rate of resistance to Sb-containing drugs (Rojo et al., 2015; Singh and Sundar, 2017; Dumetz et al., 2018). We previously employed a comparative proteomics approach to investigate global cellular Sb(III) responses in the heterotrophic Sb(III)-oxidizing *A. tumefaciens* strain, GW4 (Li et al., 2017b), and found that disproportionately more proteins involved in Sb(III) resistance and oxidation, as well as stress response, phosphate and phosphonate metabolism, carbohydrate metabolism, and amino acid metabolism were produced following Sb(III) exposure (Li et al., 2017b). While informative, proteomic information by itself cannot fully account for changes in the pool of cellular metabolites underlying metabolic phenotypes during Sb exposure. Thus, the present study was conducted to complement previous proteomics analyses and to identify important metabolic phenotypes that result from Sb exposure. Based on a comprehensive analysis of proteomics and metabolomics data, including targeted gene transcript results to confirm key observations, we provide evidence that Sb(III) initiates global stress responses that significantly impact multiple metabolic pathways. These findings provide a more complete picture of Sb(III) exposure and response, and help explain how bacteria deal with the associated increase in harmful metabolic byproducts, such as ROS. Finally, we identified metabolic pathways most impacted by Sb(III) exposure, thereby providing a novel understanding of the most relevant bacterial phenotypes.

## Materials and Methods

### Chemicals and Reagents

Where needed, we used HPLC grade methanol and acetonitrile (Tedia Co., Inc.; Fairfield, OH, United States), *LCMS* grade formic acid (Sigma-Aldrich Corp.; St. Louis, MO, United States), and deionized water (Watsons Co., Ltd; Guangzhou, China). The reference standard of L-2-chlorophenylalanine was supplied by Aladdin Industrial Corporation (Shanghai, China). All other chemicals and reagents used in this study were of analytical grade and commercially available.

### Cell Culturing

Metabolomics analyses were performed on *A. tumefaciens* strain GW4 (Fan et al., 2008) to investigate the effects of Sb(III) as K_2_Sb_2_(C_4_H_2_O_6_)_2_. Overnight cultures in 200 mL of chemically defined medium (CDM) with or without the addition of 50 μM Sb(III) (Weeger et al., 1999) were generated in five replicates each, mimicking conditions and sampling times for previously described proteomics analyses (Li et al., 2017b). After 32 hours of aerobic cultivation, bacterial cells were collected (6,000 g, 10 min, at 4°C) and rapidly washed twice with ice cold physiological saline. Each sample contained ∼10^7^ cells and pellets were stored at −80°C until metabolite extraction.

### Metabolite Extraction

Metabolites were extracted from pellets using methanol-water (cold) methods described by Rojo et al. (2015). Briefly, pellets were resuspended in 1 mL of pre-cooled methanol: water (4:1 = v:v) with the addition of 20 μL L-2-chlorophenylalanine (0.3 mg/mL) and 200 μL chloroform. Subsequently, cells were lysed on ice using ultrasonication for 20 min, and debris was centrifuged out (13,000 rpm, 15 min, at 4°C). The supernatant was collected and dried under vacuum before reconstitution in methanol:water (7:3, v:v). Following centrifugation (13,000 rpm, 15 min, at 4°C), the supernatant was filtered through a 0.22 μm nylon filter and transferred to an injection bottle for metabolite identification by LCMS.

### LCMS

Metabolites were identified using ultra-high performance liquid chromatography-quadrupole time-of-flight mass spectrometry (UPLCQ-TOF/MS, Waters Corp., Milford, MA, United States), performed by Majorbio Biological Medicine Technology Co., Ltd. (Shanghai, China). Chromatographic separation was achieved using a BEH C18 HPLC column (100 mm × 2.1 mm, 1.7 μm, Waters Corp., Milford, MA, United States). The mobile phase was (A) 0.1% formic acid and (B) acetonitrile with 0.1% formic acid. The following gradient elution procedure was used: 5–20% B for 0–2 min; 20–60% B for 2–8 min; 60–100% B for 8–12 min; 100% B for 2 min; 100% to 5% B for 14–14.5 min; and followed by a 1 min hold at 5% B. The flow rate was adjusted to 0.40 mL/min and the injection volume was set to 3 μL. For mass spectrum data acquisition, signals were collected in both positive and negative ion scanning mode from 50 to 1000 m/z. The capillary voltage, sample injection voltage and collision voltage were 1.0 kV, 40 V and 6 eV, respectively. Source temperature and cone temperature were 120°C and 500°C, respectively.

### Data Processing and Multivariate Data Analysis

Raw data were imported into the Progenesis QI software (Waters Corp., Milford, MA, United States) to carry out preliminary calibration of peak recognition by integration and retention times. Then, the obtained data matrix was used for multivariate analysis with SIMCA-P 14.0 software (Umetrics, Ume, Sweden). Orthogonal partial least squares discriminant analysis (OPLS-DA) was conducted to quantify the global differences of metabolite profiles between control and Sb(III) treatment groups and to identify the key metabolic differences. Only metabolites with variable importance projection (VIP) value ≥ 1 and *p*-value ≤ 0.05 were considered significant. The Progenesis QI software (Waters Corp., Milford, MA, United States) database and a self-built database at Majorbio Biological Medicine Technology Co., Ltd. (Shanghai, China), were used for metabolite identification. Differentially produced metabolites were placed into KEGG pathways^1^ to identify those affected by Sb(III). The putative iron-sulfur proteins in the proteome of strain GW4 were analyzed on MetalPredator^2^ using minimal functional sites (MFS) and fragment searches (Valasatava et al., 2016).

### Targeted Gene Transcription Analysis

To further investigate the transcription of genes involved in the altered metabolic pathways after Sb(III) exposure, strain GW4 was each inoculated into 100 mL of CDM with or without the addition of 50 μM Sb(III). Bacterial cells were harvested (13,400 × g for 5 min at 4°C) after 32 h cultivation. Total RNA was extracted with Trizol reagent (Invitrogen), and subsequently reverse transcribed into cDNAs using the RevertAid First Strand cDNA Synthesis Kit (Thermo) as described previously (Wang et al., 2012; Li et al., 2015). Quantitative RT-PCR was conducted with SYBR Green PCR Master Mix (Takara) and primers listed in Supplementary Table S1. The relative gene expression ratios were calculated using the 2^–ΔΔ^ ^*CT*^ method (Livak and Schmittgen, 2001; Li et al., 2019a). Significance analysis was performed by one-way ANOVA.

### Determination of the Total NADP^+^/NADPH, Total GSH, and Reduced GSH Contents

Overnight cultures of strain GW4 was each inoculated into 5 mL of CDM medium containing 0, 50, or 100 μM Sb(III), respectively. After 32 h of aerobic cultivation, 1–10 × 10^7^ cells were harvested and washed 3 times with ice cold PBS buffer. The detection of total NADP^+^ and NADPH was performed using the NADP + /NADPH fluorometric assay kit (ab176724, Abcam) according to the manufacturer’s instructions. Fluorescence intensity was measured with a multi-mode microplate reader (Cytation 5, BioTek) at a 540 nm excitation wavelength and a 590 nm emission wavelength. Total intracellular glutathione (GSH + GSSG) and reduced glutathione (GSH) levels were measured according to the manufacturer’s instructions with a colorimetric assay kit (Invitrogen). The same number of cells were resuspended in 5% 5-sulfosalicylic acid buffer and sonicated on ice for 5 min. After centrifugation (13,400 × g for 10 min at 4°C), cell lysate samples were diluted by adding 4 volumes of assay buffer for total GSH detection. 2-vinylpyridine (0.4%, Sigma-Aldrich) was added to the cell lysate and incubated for 1 h at room temperature prior to dilution, which was used to block free GSH in the samples and determine oxidized glutathione (GSSG) content. The absorbance was measured at 405 nm and the reduced GSH content was calculated by subtracting the GSSG content from the total GSH content. Protein concentration was determined using BCA assay.

## Results and Discussion

### LCMS Identification of Sb(III) Metabolite Response

**FIGURE 1:**
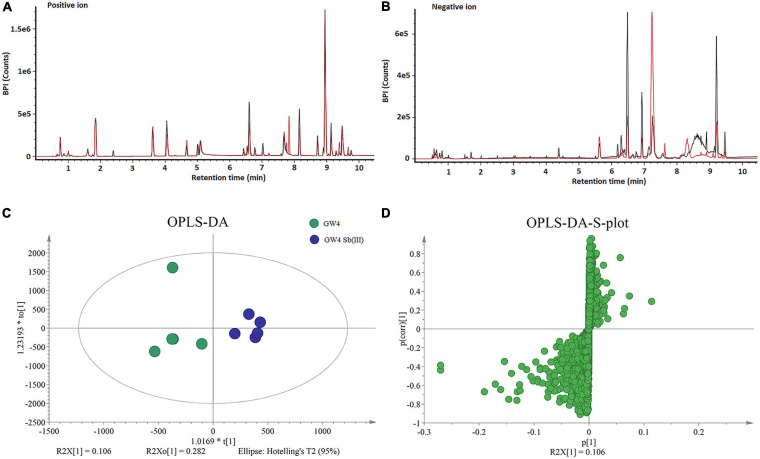
*LCMS* analysis of metabolites in strain GW4 with or without Sb(III) exposure. **(A,B)** Base peak ion chromatograms in positive **(A)** and negative **(B)** ion mode from control group (black line) and Sb(III)-treated group (red line). **(C,D)** OPLS-DA score plots and loading S-plots based on the *LCMS* data.

Five replicate cultures (i.e., biological replicates) of *A. tumefaciens* GW4 with or without the addition of 50 μM Sb(III) were used for LCMS-based metabolomics. Culture conditions and length of cultivation were identical to those described previously in our proteomics study (Li et al., 2017b). LCMS chromatograms in positive and negative ion modes (each with or without Sb(III) treatment) are shown in Figures 1A,B, respectively. No significant differences in ion retention times were observed between treatment and control groups. However, base peak intensities at the same retention time did differ for a variety of ions, supporting that metabolites production was indeed affected by Sb(III) exposure.

**FIGURE 2:**
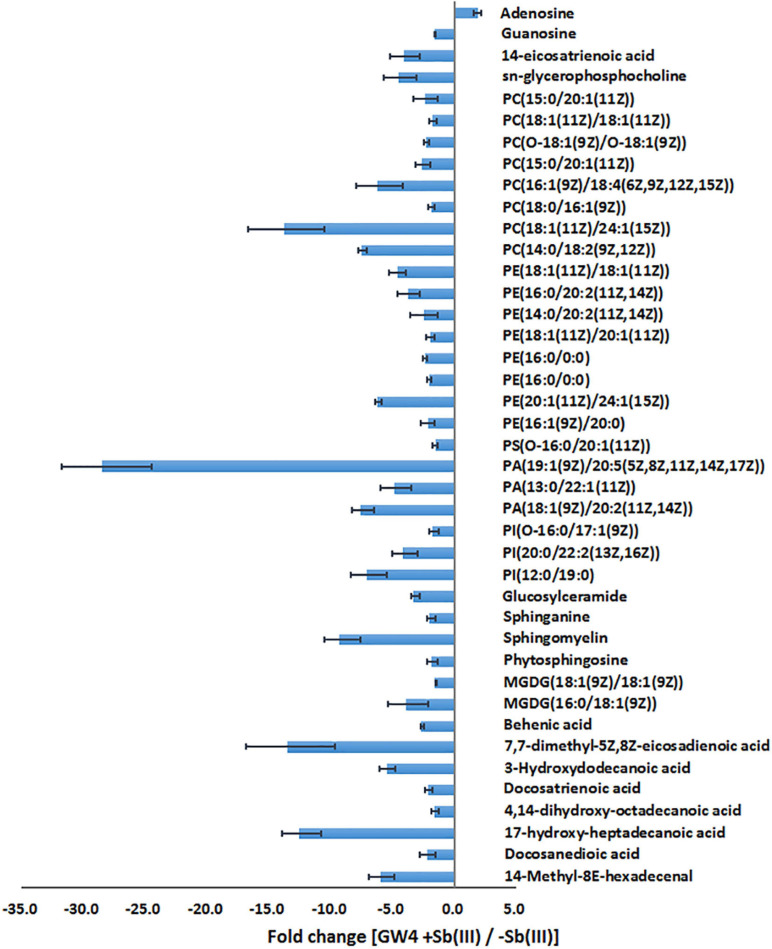
The fold change of metabolites in response to Sb(III) exposure.

Supervised orthogonal partial least squares-discriminant analysis (OPLS-DA) was used to visualize differences in metabolite profiles. OPLS-DA resulted in a good separation between Sb(III) treated and control samples (Figure 1C) and variable influence on projection (VIP) values (Figure 1D) identified metabolites most affected by Sb(III) (points farthest from the origin). A total of 170 metabolites were identified, among which two disappeared almost entirely following Sb(III) exposure. Of all 170 differentially abundant metabolites, 41 reached statistical significance (VIP value ≥ 1.0, P value ≤ 0.05; Supplementary Table S2), ranging from 3- to 30- fold change during Sb(III) exposure (Figure 2).

### Validation of Sb(III) Response Pathways

**FIGURE 3:**
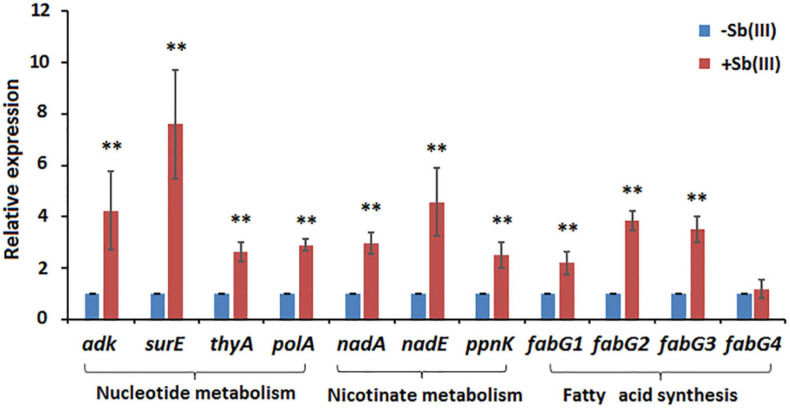
Transcription analysis of genes involved in nucleotide metabolism, nicotinate metabolism, and fatty acid synthesis by qRT-PCR. Total RNA was isolated from strain GW4 after 32 h incubation in CDM medium with or without 50 μM Sb(III). The results are shown for triplicate experiments with the error bars representing ± SD. ** represents *p* < 0.01; * represents *p* < 0.05.

Of the 41 statistically significant metabolites identified, 28 mapped to 3 different metabolic pathways (nucleotide metabolism, arachidonic acid metabolism and glycerophospholipid metabolism) (Supplementary Table S2). Interestingly, metabolites involved in the branched pathways of nucleotide metabolism, nicotinate metabolism and fatty acid synthesis were decreased during Sb(III) exposure (Supplementary Tables S2, S3). Since Sb(III) could inhibit or destroy enzymes containing an iron-sulfur (Fe-S) cluster as the catalytic center, we investigated the Fe-S proteins from the proteome of strain GW4. There was a total of 40 putative Fe-S proteins which could be mapped into KEGG pathway (Supplementary Table S4). The putative metal binding sites are shown in Supplementary Table S4. However, only one enzyme xanthine dehydrogenase, which catalyzes the reaction from hypoxanthine to xanthine, was found to be associated with the down-regulated branched pathway of nucleotide metabolism. We therefore evaluated whether a sub-set of genes in the alternate branches of these pathways were upregulated using RT-qPCR (Figure 3). Genes involved in purine synthesis (adenylate kinase gene *adk*), pyrimidine synthesis (5-nucleotidase *surE*, thymidylate synthase gene *thyA*, and DNA polymerase I gene *polA*), nicotinate metabolism (quinolinate synthetase gene *nadA*, NAD synthetase gene *nadE*, and NAD(P) transhydrogenase gene *ppnK*), and fatty acid synthesis (3-oxoacyl-[acyl-carrier protein] reductase gene *fabG*) were all upregulated by Sb(III) (Figure 3), suggesting that the response to Sb(III) had indeed switched branches in these pathways. With respect to fatty acid synthesis, three of the four *fabG* genes present in the genome of strain GW4 (*fabG1*, *fabG2*, *fabG3*) were each significantly upregulated during Sb(III) exposure, whereas the transcription of *fabG4* was not (Figure 3), indicating that only three *fabG* genes play important roles in fatty acid synthesis in strain GW4, at least under these conditions.

### Sb(III) Exposure Increases Nucleotide Synthesis

**FIGURE 4:**
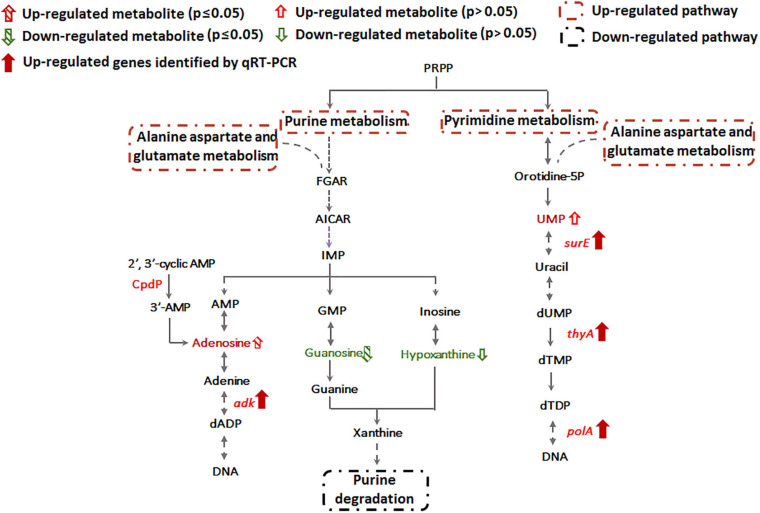
Purine and pyrimidine metabolism pathways affected by Sb(III).

Metabolomics data showed that guanosine and hypoxanthine were decreased in abundance, indicating that the purine degradation was inhibited during Sb(III) exposure (Figure 4). In contrast, we found that adenosine, which is involved in purine synthesis, increased as was transcription of the adenylate kinase gene, *adk* (Figures 3, 4). In addition, the increased accumulation of uridine 5′-monophosphate (UMP) suggests that pyrimidine metabolism was also induced by Sb(III) (Figure 4), which was further supported by increased transcription of key genes in the pyrimidine synthesis pathway, *surE*, *thyA* and *polA* (Figures 3, 4). These results provide strong support that nucleotide synthesis was activated under Sb(III) stress, possibly to repair well-known DNA damaging ROS generation (Hardy and Chaconas, 2013; Bourret et al., 2016).

### Sb(III) Exposure Shifts Nicotinate Metabolism

**FIGURE 5:**
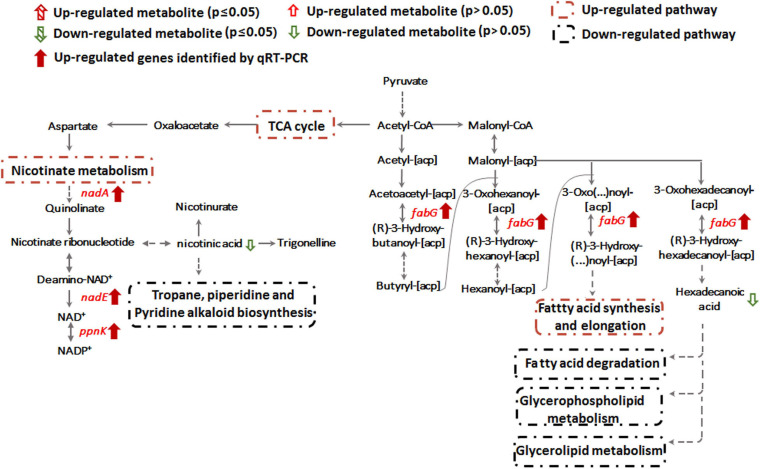
Nicotinate metabolism and lipid metabolism pathways affected by Sb(III).

**FIGURE 6:**
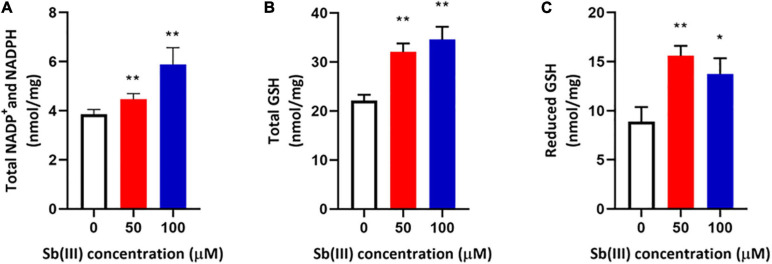
Total NADP^+^/NADPH **(A)**, total glutathione (GSH + GSSG) **(B)**, and reduced glutathione (GSH) **(C)** contents in strain GW4 after Sb(III) exposure. Bacterial cells were each inoculated into 5 mL of CDM medium containing 0, 50, or 100 μM Sb(III), respectively, and incubated at 28°C with 120 rpm shaking. After 32 h incubation, cells were harvested for different analyses. The results are shown for triplicate experiments with the error bars representing ± SD. ** represents *p* < 0.01; * represents *p* < 0.05.

Transcription of genes encoding key enzymes of NADP^+^ synthesis (*nadA*, *nadE* and *ppnK*) were induced by Sb(III) (Figures 3, 5). In addition, we found reduced nicotinic acid (Supplementary Table S3), which is involved in a branched pathway of nicotinate metabolism (Figure 5). These results suggested that aspartate generated from the TCA cycle flowed into the nicotinate metabolism pathway to produce NADP^+^ rather than into tropane, piperidine and pyridine alkaloid biosynthesis (Figure 5). As a hydride carrier, NADP^+^ plays an important role in dehydrogenase catalyzed redox reactions, such as those involving Sb(III) oxidase, AnoA, that requires NADP^+^ as a co-factor for Sb(III) oxidation (Li et al., 2015). In addition, NADP^+^ could also provide the substrate for NADPH synthesis in the pentose phosphate pathway (Moreno-Sánchez et al., 2017). To further verify the increased NADP^+^ synthesis, we detected the total content of NADP^+^ and NADPH in strain GW4. As shown in Figure 6A, the total content of NADP^+^ and NADPH was increased after Sb(III) exposure in a dose dependent manner (Figure 6A), which is consistent with the results of transcription analysis (Figure 3).

NADPH plays an important role in the oxidative stress response, which makes intuitive sense since it is an electron donor for glutathione reductase to produce reduced-GSH (Moreno-Sánchez et al., 2017). It is well known that GSH contains an active sulfhydryl (-SH) that is perhaps the most important functional group for protecting enzymes from oxidation and inactivation. Furthermore, GSH could reduce free radicals and H_2_O_2_ caused by Sb(III), thereby reducing the damage of ROS to bacterial cells (Wongsaroj et al., 2018). Consistent with these expectations, the total glutathione (GSH + GSSG) and reduced glutathione (GSH) contents were both significantly increased in strain GW4 after 50 or 100 μM Sb(III) exposure (Figures 6B,C). A 2-fold increase of GSH was detected following the exposure to 50 μM Sb(III) (Figure 6C). Based on the metabolomics data, the GSSG was only 1.5-fold increase in the presence of 50 μM Sb(III) (Supplementary Table S3), indicating that GSH contributed to ROS resistance, it was not over-oxidized in the presence of NADPH.

### Sb(III) Induces Alteration of Cell Membrane Lipids

**FIGURE 7:**
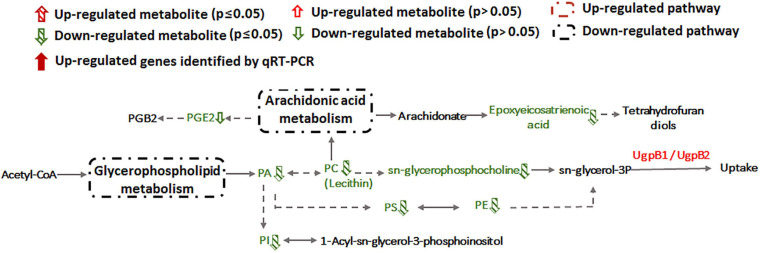
Glycerophospholipid metabolism and arachidonic acid metabolism pathways affected by Sb(III).

Alteration of lipid metabolism has been observed in cellular responses to several heavy metals and metalloids, including As(III), Cr(VI) and Cd(II) (Bagchi et al., 2002; Weiss et al., 2009; Zheng et al., 2016). Lipids in cell membranes are major targets for ROS induced by toxicants, where free radicals can attack unsaturated fatty acids resulting in lipid peroxidation (Khan et al., 2018; Bernat et al., 2018). During Sb(III) exposure, the levels of key membrane lipids (glycerophospholipid, sphingolipid, glycerolipid, fatty acid, and fatty aldehydes metabolisms) were all lower compared to unexposed cells (Supplementary Table S2 and Figure 7). The most down-regulated glycerophospholipid was phosphatidyl choline (PC), followed by phosphatidyl ethanolamine (PE), phosphatidic acid (PA), phosphatidyl inositol (PI) and phosphatidyl serine (PS). Changes in fatty acid metabolism (down-regulation of behenic acid, 7,7-dimethyl-5Z, 8Z-eicosadienoic acid, 3-Hydroxydodecanoic acid, docosatrienoic acid, 4,14-dihydroxy-octadecanoic acid, 17-hydroxy-heptadecanoic acid, and hexadecanoic acid) were also consistent with altered lipid metabolism (Supplementary Tables S2, S3). For example, according to KEGG analysis, the decreased level of hexadecanoic acid is consistent with a down-regulation of fatty acid degradation, glycerophospholipid metabolism and glycerolipid metabolism (Figures 5, 7). Furthermore, increased transcription of *fabG* may contribute to the enhanced synthesis of the fatty acid, which is consistent with observations of iron resistance in siderophilic cyanobacterium, *Leptolyngbya* strain JSC-1 (Khan et al., 2018). Finally, down-regulation of glycerophospholipids coincided with decreased accumulation of sn-glycerol-3P (Figure 7), which also acts as a phosphate source for growth (Hekstra and Tommassen, 1993). Interestingly, our previous study found that the expression of periplasmic glycerol-3-phosphate-binding protein UgpB1 and UgpB2 were significantly up-regulated by Sb(III) (Li et al., 2015). As a member of the *pho* regulon, the high-affinity inorganic phosphate (P_i_) transport system, Ugp, is induced by P_i_ starvation, which could increase glycerol-3P uptake under low-P_i_ conditions (Hekstra and Tommassen, 1993). In addition, the high-affinity phosphate binding protein PstS2 was also induced by Sb(III) (Li et al., 2017b), suggesting that the toxicity of Sb(III) may further promote P_i_ deficiencies.

### Integration of Proteomic and Metabolomic Responses to Sb(III)

**FIGURE 8:**
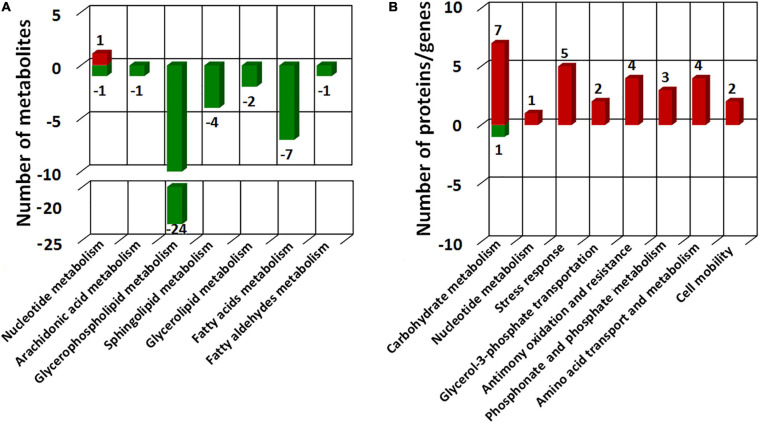
COG categories of the differential metabolites identified in this study **(A)** and different expressed proteins in our previous proteomics study (Li et al., 2015) **(B)**. Red bars represent the numbers of up-regulated metabolites/proteins and green bars represent the numbers of the down-regulated metabolites/proteins.

To comprehensively understand the potential mechanisms of cellular response to Sb(III) exposure, we compared changes observed between our metabolomics and proteomics datasets. Key metabolites identified during exposure belonged to nucleotide metabolism, arachidonic acid metabolism, glycerophospholipid metabolism, sphingolipid metabolism, glycerolipid metabolism, fatty acids metabolism, and fatty aldehydes metabolism (Figure 8 and Supplementary Table S2), whereas key proteins identified in our previous study mainly belonged to carbohydrate metabolism, nucleotide metabolism, stress response, glycerol-3-phosphate transportation, antimony oxidation and resistance, phosphonate and phosphate metabolism, amino acid transport and metabolism, and cell mobility (Li et al., 2015; Li et al., 2017b). The different pathways identified in these datasets were undoubtedly due to sensitivities inherent with the different approaches but together they provided both complementary (overlapping) as well as unique (non-overlapping) information.

**FIGURE 9:**
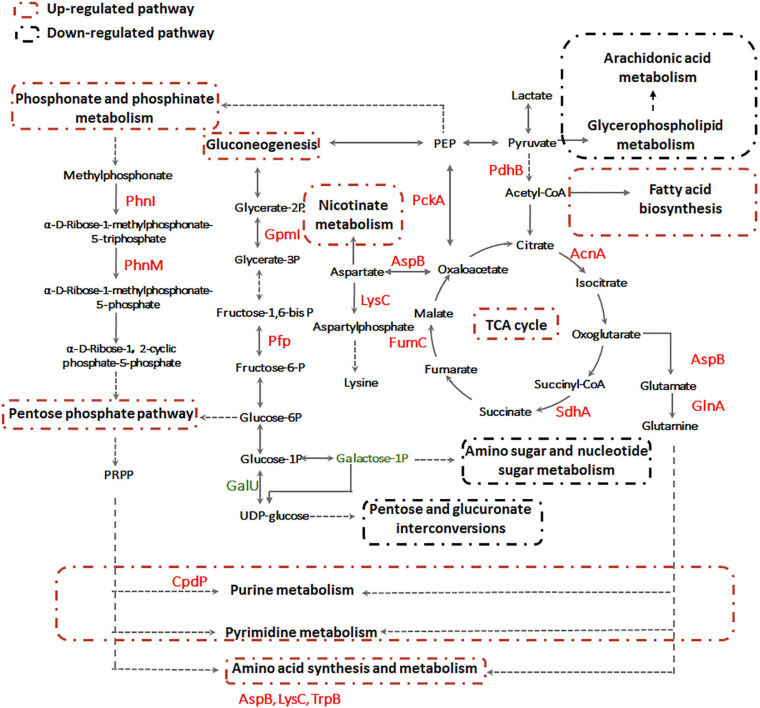
The overall metabolic pathways affected by Sb(III) in *A. tumefaciens* GW4 combination metabolomics with proteomics data.

Previously, we already identified essential genes associated with Sb(III) resistance and oxidation in strain GW4, such as Sb(III) oxidase gene *anoA*; As(III) oxidase gene *aioA*, which is also responsible for Sb(III) oxidation; catalase gene *katA*, which is related to non-enzymatic Sb(III) oxidation and Pi two-component system genes *phoBR*, which regulate the transcription of *anoA* (Li et al., 2015; Li et al., 2017a; Li et al., 2019b). In this study, by analyzing the datasets of proteomics, metabolomics and targeted gene transcription analysis, the proteins, metabolites and genes in the metabolic pathways are shown, and an overall mechanism of microbial responses to Sb(III) is established (Figure 9). Carbohydrate metabolism starts with lactate, which is the carbon source in CDM medium (Weeger et al., 1999). Both proteomics and metabolomics suggested the generated acetyl-CoA from lactate flowed into the TCA cycle for energy production and into fatty acid biosynthesis for protection from membrane lipid peroxidation, rather than glycerophospholipid metabolism. The intermediate products of TCA cycle, such as aspartate and glutamine also contributed to nicotinate metabolism, nucleotide and amino acid metabolism, respectively (Figure 9). Proteins involved in the synthesis and metabolism of various amino acids were identified in the presence of Sb(III) (Li et al., 2015; Li et al., 2017b), suggesting that the protein synthesis was increased to combat the toxicity of Sb(III) and ROS in strain GW4. Nucleotide synthesis was increased by Sb(III) exposure, which was further supported by the increased production of 35′,55′-cyclic-nucleotide phosphodiesterase CpdP, which product is another source of adenosine (Figure 4).

In addition, proteins involved in gluconeogenesis and phosphonate and phosphinate metabolism were induced by Sb(III) (Li et al., 2015; Li et al., 2017b), resulting in the up-regulation of pentose phosphate pathway (Figure 9). In contrast, the expression of UTP-glucose-1-phosphate uridylyltransferase GalU, which transforms the glucose-1P to UDP-glucose, was down-regulated (Li et al., 2017b). This is consistent with the observation in the present study that the galactose-1P was decreased in abundance after Sb(III) exposure (Supplementary Table S3). These results suggest that the glucose-6P generated from gluconeogenesis pathway flowed into the pentose phosphate pathway rather than pentose and glucuronate interconversions and amino sugar and nucleotide sugar metabolism (Figure 9). The pentose phosphate pathway plays an important role in bacterial oxidative stress response induced by Sb(III), since it could produce phosphoribosyl pyrophosphate (PRPP) and NADPH as the reducing equivalents for various biosynthesis reactions within cells (Kuehne et al., 2015). The generated PRPP is also an important precursor of nucleotide *de novo* synthesis, salvage synthesis and amino acid metabolism (Fasullo and Endres, 2015).

## Conclusion

This study is the first attempt to comprehensively investigate the global responses for microbe–Sb interaction at different levels of molecular response (mRNA, protein, and metabolite). Such complementary datasets provide a better understanding of how bacteria respond to perturbations, such as toxic Sb(III) exposure. Based on our findings in the highly Sb(III) resistant and Sb(III) oxidizing strain GW4, we conclude the following key events take place upon Sb(III) exposure: (1) Sb(III) oxidation and transport are rapidly increased to reduce toxicity; (2) pentose phosphate pathway is up-regulated to produce PRPP and NADPH; (3) macromolecular biosynthesis is increased (nucleotides, amino acids and lipids) to repair damage; (4) co-factor synthesis (nicotinate metabolism/NADP^+^) is activated for required enzymatic function (e.g., Sb(III) oxidase AnoA); and (5) TCA cycle is up-regulated to provide more energy for bacterial growth and Sb(III) resistance. Together, these results provide a much more detailed explanation of how bacteria deal with Sb(III) exposures. We anticipate this information will be critical for future studies that employ either natural or genetically modified microorganisms as Sb(III) mitigation strategies.

## Data Availability Statement

The raw data supporting the conclusions of this article will be made available by the authors, without undue reservation.

## Author Contributions

JL designed and performed the experiments and drafted the manuscript. YZ and XW performed the experiments. SW and GW designed the study and revised the manuscript. All authors read and approved the final manuscript.

## Conflict of Interest

The authors declare that the research was conducted in the absence of any commercial or financial relationships that could be construed as a potential conflict of interest.

## Footnotes

1 www.genome.jp/kegg/

2 http://metalweb.cerm.unifi.it/tools/metalpredator/

## Supplementary Material

Supplementary Figure 1 Growth **(A)** and Sb(III) oxidation curves **(B)** of strain GW4 with or without the addition of 50 μM Sb(III) in CDM medium. The Sb(V) content was measured by HPLC-HG-AFS. The arrow indicates the sampling time for metabolomics, which is consistent with our previous proteomics study (Li et al., 2015).

Supplementary Table 1 Primers used for the quantitative RT-PCR.

Supplementary Table 2 Metabolites identified using *LCMS.*

Supplementary Table 3 Metabolites which not significantly affected by Sb(III), but may also contribute to Sb(III) resistance.

Supplementary Table 4 Putative iron–sulfur proteins in the proteome of strain GW4.
