# Supplementary material for: Integrated Metabolomics and Targeted Gene Transcription Analysis Reveal Global Bacterial Antimonite Resistance Mechanisms
Source: Front Microbiol. 2021 Jan 28;12:617050. doi: 10.3389/fmicb.2021.617050 (PMC7876068; doi:10.3389/fmicb.2021.617050)
Supplement: Supplementary Figure 1 — Growth (A) and Sb(III) oxidation curves (B) of strain GW4 with or without the addition of 50 μM Sb(III) in CDM medium. The Sb(V) content was measured by HPLC-HG-AFS. The arrow indicates the sampling time for metabolomics, which is consistent with our previous proteomics study (Li et al., 2015). [file Data_Sheet_1.docx]

**Integrated metabolomics and targeted gene transcription analysis reveal global bacterial antimonite resistance mechanisms**

Jingxin Li^1^, Yuxiao Zhang^1^, Xing Wang^1^, Seth T. Walk^2^*, Gejiao Wang^1^*

**Table S1.** Primers used for the quantitative RT-PCR

**
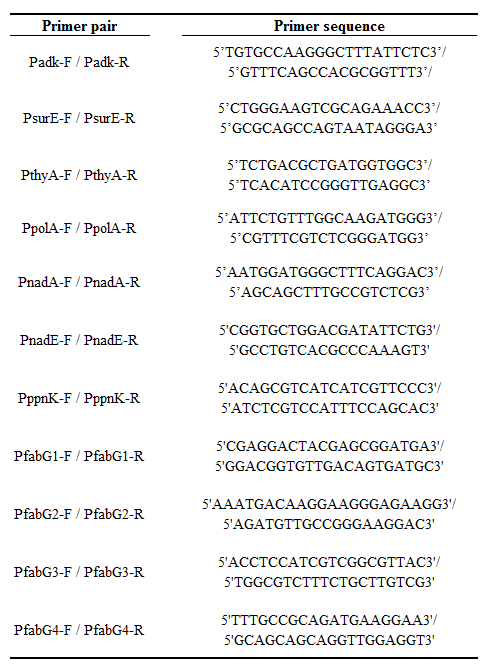
**

**Table S2.** Metabolites identified using *LCMS*

**
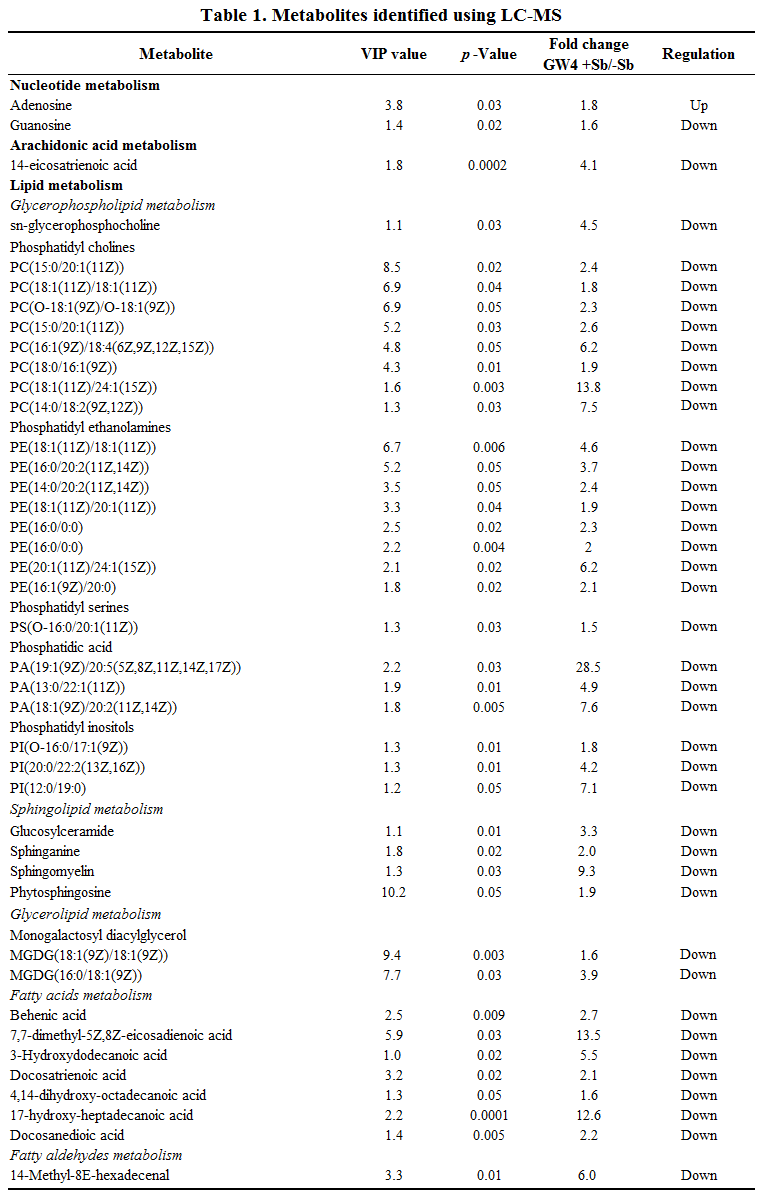
**

**Table S3.** Metabolites which not significantly affected by Sb(III), but may also contribute to Sb(III) resistance

**
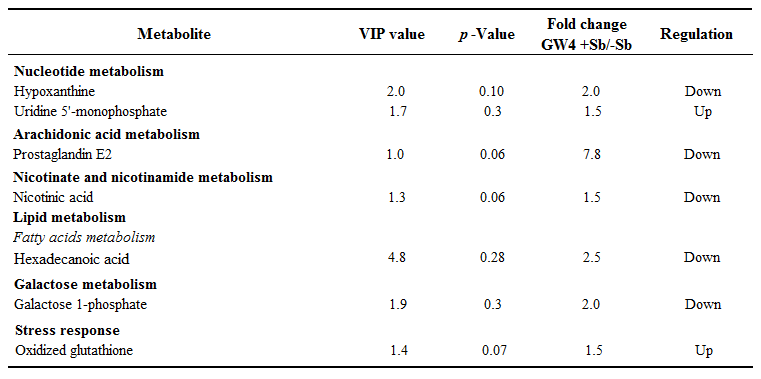
**

**Table S4.** Putative iron-sulfur proteins in the proteome of strain GW4

**
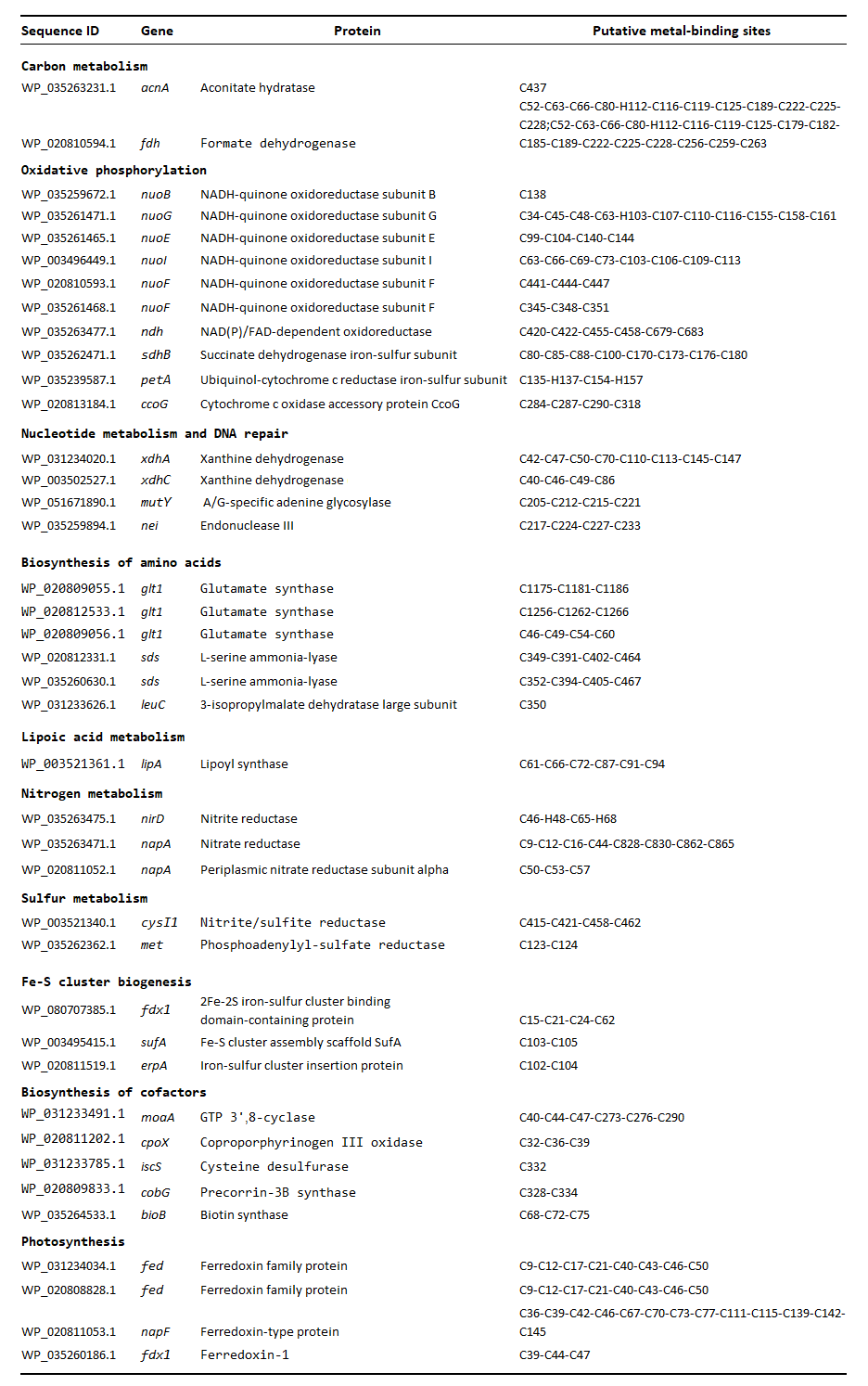
**

**
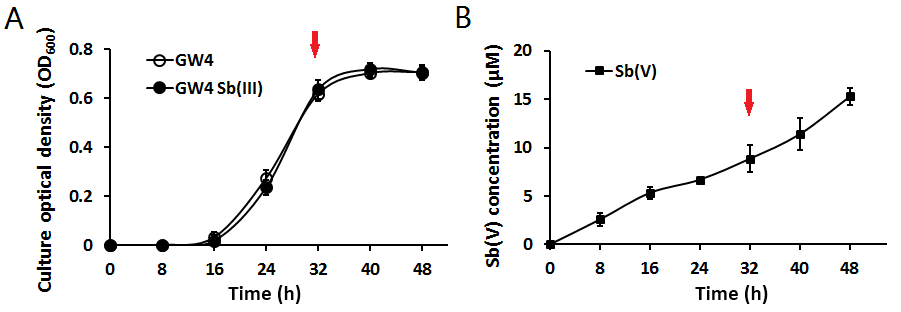
**

**Figure S1**. Growth (A) and Sb(III) oxidation curves (B) of strain GW4 with or without the addition of 50 μM Sb(III) in CDM medium. The Sb(V) content was measured by HPLC-HG-AFS. The arrow indicates the sampling time for metabolomics, which is consistent with our previous proteomics study (Li et al., 2015).
